# Giant Pleomorphic Adenoma of the Lacrimal Sac Initially Suspected to Represent a Sinonasal Malignancy 16 Years After Dacryocystorhinostomy

**DOI:** 10.3390/diagnostics16132027

**Published:** 2026-06-29

**Authors:** Shintaro Mitamura, Shunichi Oide, Yuki Sasaki, Hiroshi Idogawa, Mizuho Mitamura, Hiromi Kanno-Okada, Masayuki Osawa, Taku Maeda

**Affiliations:** 1 Department of Plastic and Reconstructive Surgery, Faculty of Medicine and Graduate School of Medicine, Hokkaido University, Sapporo 060-8638, Japan; shintaro.mitamura@huhp.hokudai.ac.jp (S.M.); ohshun3755@gmail.com (S.O.); sasakiyuki1120@gmail.com (Y.S.); takumaeda-prs@med.hokudai.ac.jp (T.M.); 2 Department of Otolaryngology-Head and Neck Surgery, Faculty of Medicine and Graduate School of Medicine, Hokkaido University, Sapporo 060-8638, Japan; charlotte69@live.jp; 3 Department of Ophthalmology, Faculty of Medicine and Graduate School of Medicine, Hokkaido University, Sapporo 060-8638, Japan; 4 Department of Surgical Pathology, Hokkaido University Hospital, Sapporo 060-8648, Japan; kanno-kanno-42010063@ion.ocn.ne.jp; 5 Department of Plastic and Reconstructive Surgery, Teine Keijinkai Hospital, Sapporo 006-8555, Japan; osw@fb3.so-net.ne.jp

**Keywords:** case report, dacryocystorhinostomy, diagnostic pitfall, lacrimal sac tumor, orbital reconstruction, pleomorphic adenoma

## Abstract

**Background and Clinical Significance**: Tumors of the lacrimal drainage system are rare but clinically important diagnostic pitfalls because they may mimic benign lacrimal drainage obstruction, chronic dacryocystitis, or sinonasal disease. Pleomorphic adenoma (PA) in this region is exceptionally rare, and delayed recognition may allow progressive extension into adjacent structures. **Case Presentation**: A 51-year-old woman presented with left nasal obstruction 16 years after external dacryocystorhinostomy for presumed lacrimal drainage obstruction. Examination showed a firm left lacrimal sac mass, proptosis, mild ocular motility limitation, and an intranasal mass. Computed tomography and magnetic resonance imaging showed a large lesion centered in the lacrimal sac region and extending into the nasolacrimal duct, nasal cavity, and maxillary sinus, with orbital displacement. Preoperative biopsy showed epithelial neoplastic tissue without definitive malignant features, but low-grade epithelial malignancy could not be excluded. Complete *en bloc* excision and medial/inferior orbital wall reconstruction with an autologous calvarial outer table bone graft were performed. The tumor measured 55 × 35 × 25 mm. Final histopathology confirmed PA without malignant transformation. At 2 years postoperatively, there was no recurrence, and proptosis and ocular motility limitation had improved. **Conclusions**: This case illustrates two diagnostic pitfalls: an underlying tumor may masquerade as lacrimal drainage obstruction, whereas a large benign PA may clinically and radiologically mimic malignancy. Long-standing or atypical unilateral lacrimal symptoms should prompt consideration of tumors and selected use of imaging and tissue diagnosis before lacrimal drainage surgery.

## 1. Introduction

Tumors of the lacrimal sac and nasolacrimal drainage system are uncommon but clinically important diagnostic pitfalls. They may present with nonspecific obstructive or inflammatory symptoms, including epiphora, discharge, or dacryocystitis-like episodes, and may therefore be mistaken for benign lacrimal drainage obstruction or chronic dacryocystitis [1,2,3,4,5]. Because lacrimal sac tumors include a broad spectrum of epithelial, nonepithelial, lymphoid, melanocytic, and other lesions, and because a substantial proportion are malignant, delayed recognition can affect diagnostic strategy and surgical management [1,2].

Diagnostic assessment can be particularly challenging when a lacrimal drainage system tumor extends beyond the lacrimal sac region into the nasal cavity, paranasal sinuses, or orbit. In such cases, clinical examination, nasal endoscopy, cross-sectional imaging, and histopathologic assessment must be integrated to distinguish lacrimal drainage obstruction, chronic inflammatory disease, sinonasal tumors, and lacrimal drainage system tumors. Pleomorphic adenoma (PA) is a well-recognized epithelial tumor of the lacrimal gland, but occurrence in the lacrimal sac or nasolacrimal duct is exceptionally rare. Subepithelial serous and mucous glands in the lacrimal sac and nasolacrimal duct walls have been proposed as a possible origin for glandular tumors in this region [6].

Here, we report a giant PA of the lacrimal sac presenting 16 years after external dacryocystorhinostomy (DCR). The lesion extended into the nasolacrimal duct, nasal cavity, and maxillary sinus, produced orbital manifestations, and was initially suspected clinically and radiologically to represent a sinonasal malignancy. This case highlights the need for multidisciplinary diagnostic assessment, including imaging, tissue diagnosis, and pathologic confirmation, and illustrates how diagnostic uncertainty can influence surgical planning, complete excision, and reconstruction.

## 2. Case Presentation

### 2.1. Clinical Presentation and Imaging Findings

**Figure 1:**
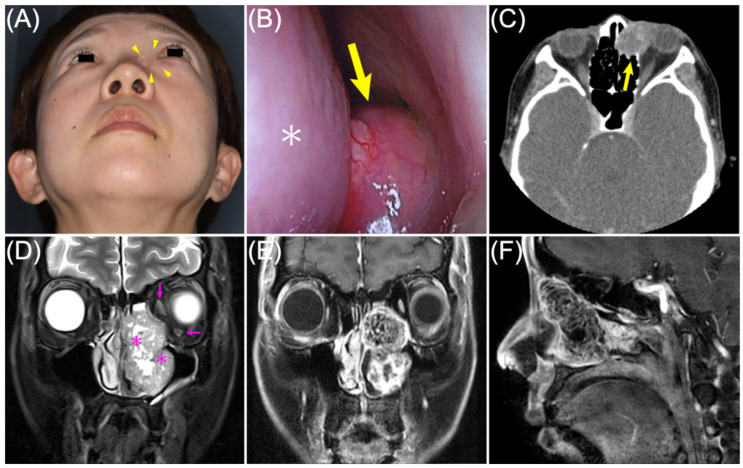
Preoperative clinical, endoscopic, and radiologic findings suggesting a large lacrimal drainage system tumor. (**A**) Upward-view facial photograph showing left-sided proptosis and a firm mass in the left medial canthal/lacrimal sac region, indicated by yellow arrowheads; (**B**) Nasal endoscopy showing an intranasal mass (yellow arrow) adjacent to the middle turbinate (white asterisk); (**C**) Contrast-enhanced computed tomography at the lacrimal sac level showing a mass centered in the left lacrimal sac region (yellow arrow); (**D**) Coronal T2-weighted magnetic resonance imaging showing a large mass extending from the lacrimal sac region into the nasal cavity and maxillary sinus. The extraocular muscles are displaced (magenta arrows), and flow voids are visible within the tumor (magenta asterisks); (**E**) Coronal gadolinium-enhanced T1-weighted magnetic resonance imaging showing heterogeneous enhancement of the lesion; (**F**) Sagittal gadolinium-enhanced T1-weighted magnetic resonance imaging showing the craniocaudal and anteroposterior extent of the lesion.

A 51-year-old woman was referred to our hospital for evaluation of a left maxillary sinus mass after presenting with left nasal obstruction. Sixteen years earlier, she had undergone external DCR for presumed lacrimal drainage obstruction. According to the patient, marked swelling had occurred after that surgery. The available records did not allow determination of whether a tumor had been present at that time. At presentation to our department, an elastic firm mass was palpable in the left medial canthal/lacrimal sac region. Hertel exophthalmometry showed 5 mm of left-sided proptosis relative to the right side. Mild limitation of superolateral gaze of the left eye was noted, which was anatomically consistent with the inferomedial location of the tumor. Nasal endoscopy showed an intranasal mass (Figure 1). Contrast-enhanced computed tomography (CT) showed a mass centered at the left lacrimal sac level. Magnetic resonance imaging (MRI) demonstrated a large lesion extending from the lacrimal sac region into the nasolacrimal duct, nasal cavity, and maxillary sinus, with displacement of the orbital contents. The lesion showed heterogeneous enhancement, and flow voids were observed. Radiologic differential diagnoses included a low-grade malignant tumor and a hypervascular tumor.

### 2.2. Biopsy Findings and Diagnostic Considerations

A preoperative endoscopic biopsy of the intranasal component performed at our institution showed epithelial neoplastic tissue. Frozen-section examination did not show marked cytologic atypia but could not exclude low-grade epithelial malignancy because the limited biopsy specimen did not permit assessment of the entire lesion. Permanent sections showed no definitive malignant features; however, definitive histopathologic characterization required examination of the complete resection specimen. Based on the imaging and biopsy findings, the lesion was initially managed as a suspected sinonasal malignancy.

Because of the large tumor size, adjacent extension, long clinical interval, preoperative biopsy findings that could not exclude epithelial malignancy, and radiologic concern for a low-grade malignant tumor, carcinoma ex pleomorphic adenoma (CXPA) could not be completely excluded. Complete *en bloc* excision and reconstruction were planned as a multidisciplinary procedure.

### 2.3. En Bloc Excision and Orbital Reconstruction

**Figure 2:**
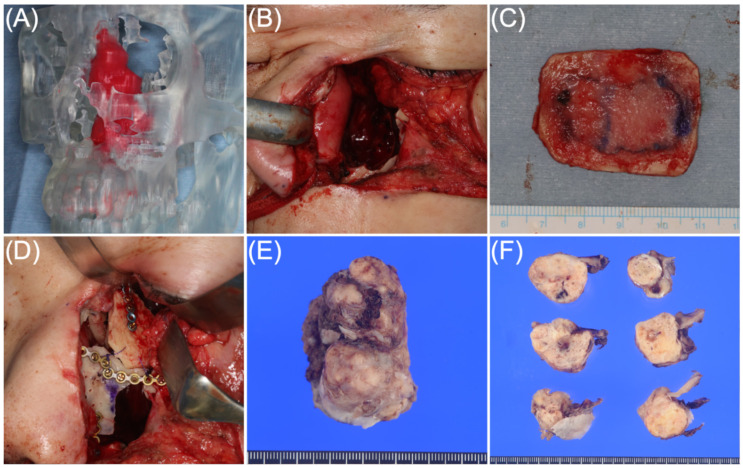
Surgical planning, complete excision, reconstruction, and gross specimen. (**A**) Three-dimensional printed surgical model used for preoperative visualization of the tumor–bone relationship and osteotomy planning; (**B**) Intraoperative view through a Weber incision showing the surgical defect after *en bloc* excision, including focal bony defects associated with pressure-related remodeling; (**C**) A 4.0 × 3.5 cm autologous calvarial outer table bone graft harvested for orbital reconstruction; (**D**) Reconstruction of the medial and inferior orbital wall using the calvarial bone graft, with medial canthal tendon fixation by transnasal wiring; (**E**) Gross specimen viewed from the cutaneous/body-surface side; the superior aspect corresponds to the cranial side and the inferior aspect to the caudal side. The tumor measured 55 × 35 × 25 mm; (**F**) Cut surfaces of the resected specimen showing a solid, lobulated, yellowish-white tumor with a relatively well-demarcated gross appearance.

Preoperative 3D surgical simulation was performed using CT data in Digital Imaging and Communications in Medicine format imported into Mimics Innovation Suite, version 26 (Materialise, Leuven, Belgium). A 3D-printed model was created to visualize the tumor–bone relationship and plan the osteotomy. The surgical plan was to temporarily remove the anterior bony wall overlying the tumor, achieve *en bloc* excision, reposition the bone segment, and reconstruct the residual orbital defect using an autologous calvarial outer table bone graft. Surgery was performed jointly by the Departments of Otolaryngology-Head and Neck Surgery and Plastic and Reconstructive Surgery. The superior aspect of the tumor was exposed through a coronal incision. A Weber incision and endoscopic approach were then used to control the anterior, nasal, and posterior aspects of the tumor. Complete *en bloc* excision was achieved without apparent intraoperative tumor rupture. No macroscopic residual tumor was apparent at the completion of the procedure. The surgical defect included the medial and inferior orbital wall region. A 4.0 × 3.5 cm autologous calvarial outer table bone graft was harvested and used to reconstruct the medial and inferior orbital wall. Medial canthal tendon fixation was performed using transnasal wiring. Additional lacrimal drainage reconstruction or stenting was not performed because CXPA could not be excluded preoperatively, and manipulation of the potentially tumor-involved lacrimal drainage pathway was considered to carry a theoretical oncologic risk. The resected tumor measured 55 × 35 × 25 mm (Figure 2).

### 2.4. Histopathologic Findings and Follow-Up

Histopathologic examination of the complete resection specimen showed biphasic epithelial and myoepithelial components forming small ductal structures within myxoid and hyalinized stroma. Cytokeratin 7 immunostaining showed epithelial differentiation in the ductal structures, whereas p63 immunostaining highlighted the myoepithelial component. No infiltrative growth pattern, significant cytologic atypia, increased mitotic activity, necrosis, or malignant epithelial component was identified. Separately submitted specimens from the posterior inferior turbinate, maxillary sinus, and nasal septum showed no neoplastic change. The final diagnosis was PA, with no histopathologic evidence of CXPA. Ki-67 immunostaining was not performed because the complete resection specimen showed no morphologic features suggestive of malignant transformation.

**Figure 3:**
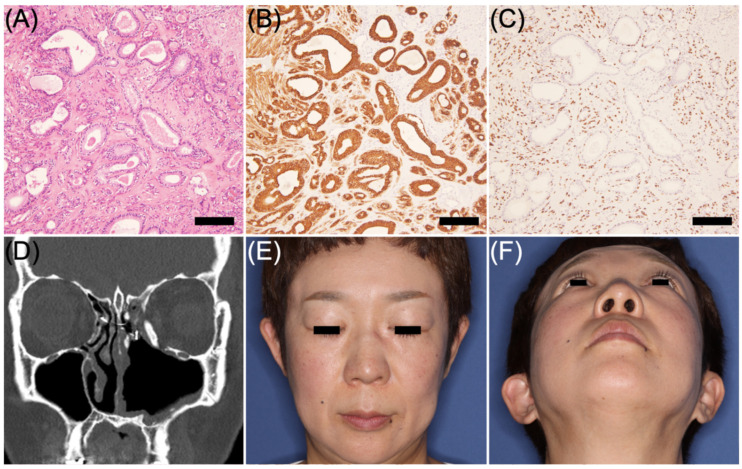
Histopathologic findings and 2-year postoperative outcome. (**A**) Hematoxylin and eosin staining showing small ductal structures within myxoid and hyalinized stroma; (**B**) Cytokeratin 7 immunostaining showing epithelial differentiation in the ductal structures; (**C**) p63 immunostaining highlighting the myoepithelial component; (**D**) Coronal computed tomography obtained 2 years postoperatively showing no recurrence and stable reconstruction of the medial and inferior orbital wall; (**E**) Frontal photograph obtained 2 years postoperatively showing acceptable cosmetic outcome; (**F**) Upward-view photograph obtained 2 years postoperatively showing improvement of left-sided proptosis. Scale bars in (**A**–**C**), 200 μm.

At 1 year postoperatively, Hertel exophthalmometry showed improvement in left-sided proptosis from 5 mm preoperatively to 1 mm. At 2 years postoperatively, CT showed no evidence of recurrence and stable orbital reconstruction (Figure 3). Ocular motility limitation had improved, and no diplopia was present (Figure S1). Mild epiphora persisted but was not bothersome to the patient, and she did not wish to undergo secondary lacrimal drainage reconstruction. No discharge or recurrent dacryocystitis-like symptoms occurred during follow-up.

The institutional review board of Hokkaido University Hospital waived ethical assessment because this was a single case report. The study adhered to the tenets of the Declaration of Helsinki. Written informed consent for publication, including clinical images and radiologic/pathologic data, was obtained from the patient after detailed explanation.

## 3. Discussion

This case illustrates a diagnostic pitfall in the evaluation of a large lacrimal drainage system tumor that was initially suspected clinically and radiologically to represent a sinonasal malignancy. The tumor was centered in the lacrimal sac region but extended into the nasolacrimal duct, nasal cavity, and maxillary sinus, causing proptosis and mild ocular motility limitation. Preoperative biopsy showed epithelial neoplastic tissue; frozen-section examination could not exclude an epithelial malignancy, whereas permanent sections showed no definitive malignant features. In addition, the lesion was large, long-standing, and radiologically suspected to represent a low-grade malignant tumor. Thus, despite the absence of definitive malignant features on permanent biopsy sections, carcinoma ex pleomorphic adenoma (CXPA) could not be excluded preoperatively. Complete *en bloc* excision and reconstruction allowed definitive diagnosis and a favorable 2-year postoperative outcome without recurrence. This case therefore illustrates two complementary diagnostic pitfalls: the possibility that an underlying tumor may masquerade as lacrimal drainage obstruction and the possibility that a benign lacrimal sac PA may clinically and radiologically mimic malignancy when it becomes large and locally extensive.

Tumors of the lacrimal sac and nasolacrimal drainage system are uncommon but clinically important because they can mimic benign lacrimal drainage obstruction or chronic dacryocystitis. In a review of more than 400 primary lacrimal sac tumors, approximately 72% were malignant, underscoring the importance of early recognition [2]. Their symptoms are often nonspecific, including epiphora, discharge, medial canthal swelling, and recurrent dacryocystitis-like episodes, and delayed or missed diagnosis has been reported [2,3,5]. Tumors may also be encountered among patients undergoing DCR for presumed obstruction: Mihailovic et al. identified lacrimal sac tumors in 13 of 878 patients undergoing external DCR, Tanweer et al. reported nasolacrimal duct tumors exposed in 4 of 525 endoscopic DCR procedures, and a Japanese clinical review found malignant tumors in 16 of 1412 patients referred for endoscopic DCR [4,7,8]. These findings support careful diagnostic assessment rather than proceeding directly to lacrimal drainage surgery in selected patients with atypical features.

Clinical features that should raise suspicion include long-standing or atypical unilateral symptoms, a firm mass, extension beyond the lacrimal sac region, hemolacria or bloody discharge, nasal symptoms, and orbital manifestations such as proptosis, globe displacement, diplopia, or ocular motility disturbance [2,4,5,9]. In such selected patients, CT or MRI should be considered before lacrimal drainage surgery because imaging can define the three-dimensional relationship between the lesion and surrounding bony and soft-tissue structures and can help determine whether the lesion is confined to the lacrimal drainage system or extends into the nasal cavity, paranasal sinuses, or orbit [4]. Jones historically classified lacrimal sac tumors into four clinical stages: tearing alone, simulated dacryocystitis, painless nonreducible swelling in the lacrimal sac region, and extension outside the sac [3]. The present case corresponded to the fourth stage, illustrating that delayed recognition can allow local progression and make excision and reconstruction more extensive.

The temporal relationship between the previous DCR and the present tumor should be interpreted cautiously. Detailed records of the preoperative symptoms, imaging findings, operative findings, and histopathology from the earlier DCR were unavailable; therefore, it cannot be determined whether the tumor was already present at the time of the initial surgery or developed later. Unilateral lacrimal symptoms at the time of the previous treatment, the patient’s recollection of marked postoperative swelling, and subsequent ipsilateral recurrence after a long interval raise, but do not establish, the possibility that an underlying tumor was already present at the time of the initial DCR. This possibility remains unresolved and should not be interpreted as a confirmed delayed diagnosis.

Recurrent symptoms after DCR are more commonly caused by benign postoperative ostium problems, including membranous scarring, granulation tissue, synechiae, and inadequate bony ostium formation [10]. However, in patients with atypical unilateral features such as nasal obstruction, a firm medial canthal mass, hemolacria or bloody discharge, or orbital signs, lacrimal sac or nasolacrimal duct tumors should be considered before recurrence is attributed solely to postoperative scarring or ostium failure [2,4,5,9].

Although PA is common in the major salivary glands and is a well-recognized epithelial tumor of the lacrimal gland, involvement of the lacrimal sac or nasolacrimal duct is exceptionally rare. Pe’er et al. showed that normal serous and mucous glands may be present in the walls of both structures, providing a plausible origin for glandular tumors in this region [6]. To contextualize the diagnostic presentation and clinical management of this case, we reviewed reported PAs of the lacrimal sac and nasolacrimal drainage system (Table 1) [6,11,12,13,14]. To our knowledge, the present tumor is the largest reported PA in this region among cases with reported dimensions. McCool’s early “mixed-cell tumor” was excluded from this comparison because Pe’er et al. later noted that the lesion had been characterized as malignant [6,15].

The treatment strategy was determined by the inconclusive biopsy findings and the overall clinical context. Although permanent biopsy sections showed no definitive malignant features, frozen-section examination could not exclude epithelial malignancy, and the lesion was large, long-standing, and radiologically suspected to represent a low-grade malignant tumor. Therefore, CXPA could not be excluded preoperatively. Because CXPA has been reported in the lacrimal sac and nasolacrimal duct, malignant transformation should be considered in long-standing lesions in this anatomical region [16]. Complete *en bloc* excision was therefore performed for both therapeutic and definitive diagnostic purposes.

Although DCR was performed in several previously reported cases, we intentionally avoided additional lacrimal drainage reconstruction or stenting because malignancy remained a preoperative concern. Instrumentation of a potentially tumor-involved lacrimal drainage pathway was judged to carry a theoretical oncologic risk, and *en bloc* excision was prioritized. Lacrimal sac and nasolacrimal duct tumors include a relatively high proportion of malignancies; therefore, biopsy may be necessary but should be planned with oncologic caution. The lacrimal gland PA literature supports planned complete excision and avoidance of capsular disruption, incomplete excision, or unplanned biopsy [17,18,19]. Once PA is suspected and excision is undertaken, planned complete *en bloc* excision with preservation of the capsule should be considered.

In the present case, the extent of the tumor required a multidisciplinary approach combining external and endoscopic access to achieve complete removal and reconstruction of the medial and inferior orbital wall. Autologous calvarial bone was selected for reconstruction because postoperative irradiation would have been considered if malignant transformation had been identified. Although the tumor size resulted in an extensive surgical defect, stable reconstruction, acceptable cosmesis, and resolution of ocular symptoms were achieved at 2 years postoperatively.

This report has inherent limitations as a single case report. In addition, the available clinical information cannot prove whether the tumor was present at the time of the previous DCR. Nevertheless, the case emphasizes that delayed recognition may allow progressive enlargement and extension into adjacent structures, making surgical management more complex. In long-standing lesions, CXPA should also be considered. Long-term clinical and radiologic surveillance is warranted because PA may recur after incomplete excision, and late recurrence or malignant transformation, although rare, has been described [17,18,19,20].

## 4. Conclusions

This case demonstrates that lacrimal sac and nasolacrimal duct tumors may mimic benign lacrimal drainage obstruction, chronic dacryocystitis, or sinonasal malignancy. In patients with long-standing, atypical, or unilateral lacrimal symptoms, particularly when a firm mass, nasal symptoms, orbital signs, or bloody discharge is present, CT or MRI and tissue diagnosis should be considered before lacrimal drainage surgery. When PA is suspected but malignancy cannot be excluded, multidisciplinary diagnostic and surgical planning and complete *en bloc* excision with appropriate reconstruction may allow definitive diagnosis and favorable functional and aesthetic outcomes.

## Figures and Tables

**Table 1:** Clinical and diagnostic features of reported pleomorphic adenomas of the lacrimal sac and nasolacrimal drainage system.

Author, Year	Age/Sex	Site	Presentation	Size	Treatment	Follow-Up Outcome
Pe’er, 1996 [6]	55/M; 80/M	LS	NR	NR	Excision	NR
Lee, 2015 [11]	60/M	LS	Epiphora, 5 y; bloody discharge, 1 mo	1.2 × 1.2 × 1.6 cm	Open DCR + excision	No recurrence at 1 mo
Haft, 2018 [12]	60s/M	NLD	Epiphora, 2 y; hemolacria, 6 wk	NR	Endoscopic DCR + medial maxillectomy	No recurrence at 8 wk
Faizal, 2021 [13]	41/M	NLD	Painless medial canthal swelling	NR	Complete excision with capsule	No recurrence reported; duration NR
Zhang, 2024 [14]	66/F	NLD + LS	Nasal obstruction, tearing, eyelid crusting, tongue- tip hypoesthesia	1.5 × 1.4 × 2.5 cm	Partial maxillectomy + DCR	No recurrence at 10 mo
Present case	51/F	LS → NLD	Nasal obstruction, firm LS/intranasal mass, proptosis	5.5 × 3.5 × 2.5 cm	*En bloc* excision + orbital reconstruction	No recurrence at 2 y

DCR, dacryocystorhinostomy; F, female; LS, lacrimal sac; M, male; mo, months; NLD, nasolacrimal duct; NR, not reported; PA, pleomorphic adenoma; wk, weeks; y, years. McCool’s lesion was excluded because it was later considered malignant by Pe’er et al. [6,15]. Stefanyszyn’s two benign mixed tumors are represented by Pe’er’s two PA cases [1,6]. NLD cases were included as part of the continuous nasolacrimal drainage system.

## Data Availability

Data sharing is not applicable to this article because no datasets were generated or analyzed beyond the clinical data presented in this case report.

## Supplementary Materials

The following supporting information can be downloaded at: https://www.mdpi.com/article/10.3390/diagnostics16132027/s1, Figure S1: Postoperative ocular motility findings.

## Abbreviations

The following abbreviations are used in this manuscript:

CT	computed tomography
CXPA	carcinoma ex pleomorphic adenoma
DCR	dacryocystorhinostomy
MRI	magnetic resonance imaging
PA	pleomorphic adenoma
