# Supplementary figures and images for: Giant Pleomorphic Adenoma of the Lacrimal Sac Initially Suspected to Represent a Sinonasal Malignancy 16 Years After Dacryocystorhinostomy
Source: Diagnostics (Basel). 2026 Jun 29;16(13):2027. doi: 10.3390/diagnostics16132027 (PMC13360214; doi:10.3390/diagnostics16132027)

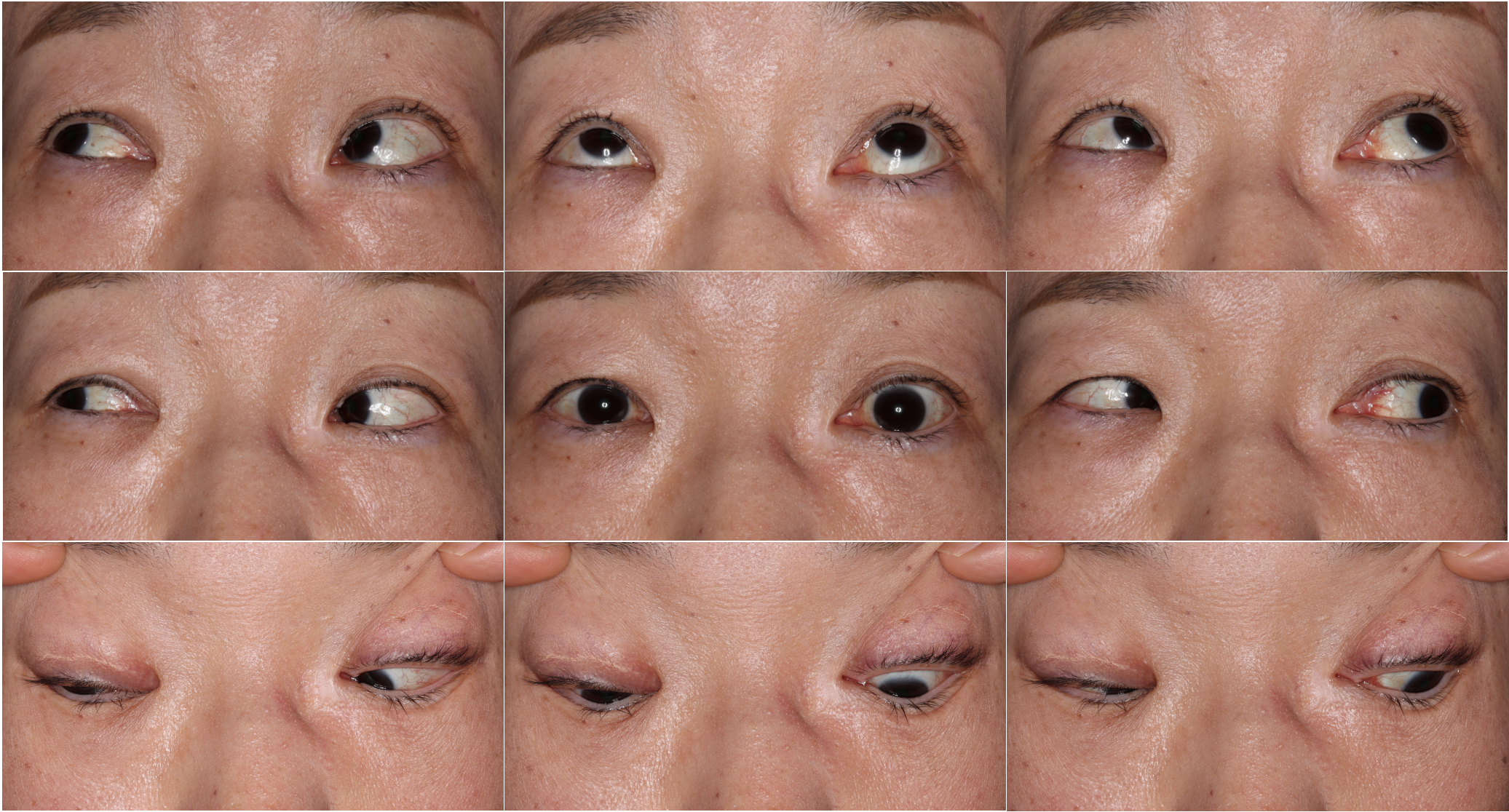

Supplement: Supplementary file 1 [file diagnostics-16-02027-s001.zip › Figure S1. Postoperative ocular motility findings.pdf]
